# The endangered common hamster needs proteins: identifying diversified crop-based diets improving hibernation and reproductive success

**DOI:** 10.1093/conphys/coae082

**Published:** 2024-12-13

**Authors:** Timothée Gérard, Jean-Patrice Robin, Florian Kletty, Caroline Habold

**Affiliations:** Université de Strasbourg, Centre National de la Recherche Scientifique, Institut Pluridisciplinaire Hubert Curien, Unité Mixte de Recherche 7178, 23 rue du Loess, 67200 Strasbourg, France; Office de Génie Écologique - O.G.E., 10 rue du Rieth 67200 Strasbourg, France; Université de Strasbourg, Centre National de la Recherche Scientifique, Institut Pluridisciplinaire Hubert Curien, Unité Mixte de Recherche 7178, 23 rue du Loess, 67200 Strasbourg, France; Université de Strasbourg, Centre National de la Recherche Scientifique, Institut Pluridisciplinaire Hubert Curien, Unité Mixte de Recherche 7178, 23 rue du Loess, 67200 Strasbourg, France; Institut Catholique de Lille, Junia, Université Catholique de Lille, Laboratoire Interdisciplinaire des Transitions de Lille, 60 boulevard Vauban, 59000 Lille, France; Université de Strasbourg, Centre National de la Recherche Scientifique, Institut Pluridisciplinaire Hubert Curien, Unité Mixte de Recherche 7178, 23 rue du Loess, 67200 Strasbourg, France

**Keywords:** Agriculture, agroecology, conservation, European hamster, torpor

## Abstract

Modern production-oriented farming has led to a decline in agricultural biodiversity. In Europe, one example of concern is the common hamster *Cricetus cricetus*, a hibernating rodent once largely distributed in farmlands and now listed as Critically Endangered. The decline of this species is tied to a significant decrease in hamsters’ body mass at emergence from hibernation and in reproduction rate. Previous work has shown that monocultures of maize and wheat, which induce severe nutrient deficiencies, are a major cause of this phenomenon. To prevent such deficiencies, we tested in controlled conditions the effect of eight nutritive diets on hamsters’ hibernation and reproduction. Diets were selected for their nutritional content and based on farmers’ consultation. We tested three lipid-rich associations (‘oat–sunflower’, ‘potato–sunflower’ and ‘buckwheat–sunflower’), two protein-rich associations (‘maize–bean’ and ‘maize–pea’) and an intermediate association (‘wheat–soybean’), as well as ‘sprouted wheat’ and ‘sugar beet’*.* Hamsters fed the lipid-rich diets showed a better body condition at the end of hibernation. However, a low reproductive success was recorded in all groups, with only 17% of reproductive trials leading to birth. Nonetheless, the amount of protein ingested by the mothers before reproduction increased birth probability whilst pups’ survival to weaning was positively correlated to mothers’ body condition. Overall, our results show that hamsters need a balanced diet to ensure their ability to reproduce. Indeed, low-protein diets led to lower birth rates and low-lipid diets led to reduced body condition and subsequently low pups’ survival. The ‘wheat–soybean’ association best fulfilled hamsters’ nutritional needs. Overall, these results highlight the need to improve current farming practices to provide a more fulfilling nutritional environment for common hamsters and the farmland fauna.

## Introduction

Biodiversity is fading at an alarming rate (Barnosky *et al.*, 2011). In Western European farmlands, this phenomenon is closely linked to the farming industrialization through the 20th century (Stoate *et al.*, 2009). Modern production-oriented farming is characterized by monoculture and high uses of chemicals (Crews *et al.*, 2016). This has led to homogenized, polluted and poorly diversified farmland habitats, threatening farmland biodiversity (Stoate *et al.*, 2009). Farmlands birds (Stanton *et al.*, 2018), insects (Sánchez-Bayo and Wyckhuys, 2019), earthworms (Blakemore, 2018) and mammals such as the common hamster *Cricetus cricetus* (Linnaeus, 1758) are severely declining (Surov *et al.*, 2016). This is threatening ecosystemic services, which in turn induces projected yield reductions and is therefore a key issue regarding agricultural production and human food security (Altieri, 1999; Schauberger *et al.*, 2018).

The common hamster is native to steppe environments and initially thrived with the development of agricultural areas and the associated food abundance. However, it is today threatened throughout its range, from the Alsace region in northeastern France to Russia (Surov *et al.*, 2016) and was recently listed as Critically Endangered on the IUCN Red List (Banaszek *et al.*, 2020). In Alsace, the decline has been particularly steep. The species has gone from pest to critically endangered in just a few decades (Kletty *et al.*, 2020). This decline was mainly caused by fur trapping and eradication policies aiming at the destruction of the species until 1993, when it was protected in France (Surov *et al.*, 2016). However, populations have since struggled to recover, despite the implementation of several conservation plans based on population reinforcement with captive-bred individuals and habitat restoration (Virion, 2018). The failure of wild populations to recover has been connected to climate change and to a degraded habitat dominated by monocultures, leading to major nutritional deficiencies, (Tissier *et al.*, 2017, 2016; Kletty, 2020). We have previously identified that a deficiency in vitamin B3 and its tryptophan precursor in maize monotonous diets resulted in reproductive failure in hamsters, with 95% of females eating their offspring (Tissier *et al.*, 2017). In wheat-dominated conditions, protein deficiencies induced a reduced reproductive success, with a lower number of pups showing low survival and growth (Tissier *et al.*, 2017, 2018). Wild females exhibit small home ranges of ~0.22 ha, several times smaller than average monocultural plots (Ulbrich and Kayser, 2004), thus preventing diet enrichment through inter-plot foraging. In the wild, the reproductive success of the common hamster is currently estimated to be 1.6 litters of 3–4 pups per year, whilst it used to be of at least 3 litters of 6–12 young in favourable years before 1950 (Surov *et al.*, 2016). This estimate applies to its entire range, not just our study area, testifying to the scale of the problem. Such low estimates explain why this prey species’ reproductive success cannot currently compensate for mortality (Nechay *et al.*, 1977; Surov *et al.*, 2016). In addition to deficiencies, monocultures cause a lack of protective cover for a significant part of the year (from July to March for wheat and October to May–June for maize; Sánchez *et al.*, 2014), further exposing common hamsters to predators during their active season (from April to September; Nechay *et al.*, 1977).

Crop–induced food availability is also a determining factor in winter survival. Common hamsters are hibernators, i.e. they decrease energy expenditure in winter by performing torpor bouts in their burrow, and periodically emerging back to euthermia to feed on the seed and tuber reserves they have accumulated before winter (Monecke and Wollnik, 2005; Hufnagl *et al.*, 2011). This food-storing hibernation strategy makes non-perishable food items availability critical for hamsters in late summer and the beginning of autumn. Energy balance during hibernation and resulting hamsters’ body mass at emergence—as a marker of body condition—are tightly linked to the quantity and quality of food hoards as well as torpor use (Siutz *et al.*, 2017; Siutz and Millesi, 2017). During hibernation, lipid-rich diets are linked to higher energy intake thus allowing a reduced use of torpor without negatively affecting body mass at emergence (Weitten *et al.*, 2018). However, a 21% decrease in the body mass of male and female hamsters at emergence has been observed in Alsace since 1937 (Tissier *et al.*, 2016). This is critical, especially for females, as hamster body condition at the end of winter is crucial for reproduction (Nechay *et al.*, 1977; Weitten *et al.*, 2018). It is therefore essential to search for crops or crop associations that are beneficial for hibernation, body condition at emergence and reproduction in female common hamsters.

In a previous study conducted in mesocosms, Tissier *et al.* (2018) showed that mixed crops (wheat, maize, alfalfa and sunflower) allow for a restoration of the hamsters’ reproductive success. In addition, Tissier *et al.* (2021) also showed that mixes such as ‘wheat–soybean’ and, to a lower extent, ‘maize–sunflower’ can enhance reproductive success. Nevertheless, a global diversification of the Alsatian farmland requires the implementation of more than two sets of crops. As part of the conservation plan for the species, the Alsatian agricultural agency and farmers were consulted. This allowed to identify technical and economic constraints shaping farming practices in the region. Crop associations were subsequently designed as viable to be implemented, either as mixed cropping, relay cropping or crop rotation. Farmers’ consultation confirmed the necessity to complement crops of high economic values, such as wheat (*Triticum sp.*), maize (*Zea mays*) and sunflower (*Helianthus annuus*), or to test the suitability of sugar beet (*Beta vulgaris*) for hamsters. These crops were tested in association with other crops of nutritional interest and agronomical relevance: soybean (*Glycine max*), oat (*Avena sativa*), buckwheat (*Fagopyrum esculentum*), potatoes (*Solanum tuberosum*), bean (*Phaseolus vulgaris*) and pea (*Pisum sativum*). ‘Maize’ was associated with ‘bean’ or ‘pea’, two legumes compensating for maize deficiencies in proteins. ‘Wheat’ was associated with ‘soybean’. This association was previously identified as nutritious for hamsters and is the positive control in our experiment (Tissier *et al.*, 2021). ‘Sunflower’, which has very low carbohydrate content, was supplemented with carbohydrate-rich ‘potatoes’*,* ‘oat’ or ‘buckwheat’. ‘Sugar beet’ was tested alone, as its interest had never been evaluated before and because it is especially difficult to couple with other species in the field. Finally, a group was fed ‘sprouted wheat’ to evaluate if sprouting can improve the limited suitability of wheat diet for hamsters. Indeed, crop or weed seeds can sprout in hamsters’ burrows and may be favourable through an increased nutrient bioavailability (Gunathunga *et al.*, 2024).

Hamsters were monitored in laboratory-controlled conditions during hibernation and reproduction. They were fed with seeds or tubers from the tested crops, which they usually consume during hibernation and at the beginning of the breeding season in the wild. Due to tuber conservation issues, the ‘sugar beet’ diet was only provided during hibernation and switched to ‘wheat–soybean’ for reproduction. We predicted that (1.a) cultural associations allowing for a higher lipid input (‘oat–sunflower’, ‘potato–sunflower’, ‘buckwheat–sunflower’ and ‘wheat–soybean’) would induce a reduced time spent in torpor in favour of activity, allowing more food ingestion. (1.b) This effect would be linked to a better body condition at the end of hibernation as described by Tissier *et al.* (2021). (1.c) Hamsters showing a better body condition at the end of hibernation would have a higher reproductive success. (2) Reproductive success will directly benefit from crop associations that provide a higher amount of proteins during the reproduction period (‘maize–bean’, ‘maize–pea’ and ‘wheat–soybean’).

## Materials and Methods

### Ethics

The study followed the European Directive 2010/63/EU on the protection of animals used for scientific purposes, and was approved by the Ethical Committee (CREMEAS) and the French Ministère de l’Enseignement Supérieur et de la Recherche (MESR) under agreement number 00624–01 on 29 November 2013 and renewed on 2 April 2019, under APAFIS# agreement 17 484–2 018 103 016 124 862 v3.

### Hamster housing conditions and diets

The study was conducted in controlled laboratory conditions on 102 hamsters (51 males and 51 females) all ~1 year old, previously fed with a conventional diet (pellets 105, from Safe, Augy, France, composed of 19.3% protein, 54.9% carbohydrates, 5.1% lipids, 4.2% cellulose, 5.0% minerals and 11.5% water). The tested diets were designed with regards to documented macronutrient content of the crops and hamsters’ known nutritional needs as well as agrotechnical constraints depicted by farmers (Feedipedia—Heuzé *et al.*, 2017). The macronutrient and energy contents of the diets were quantified afterwards (see below) and are presented in Supplementary Table S1. On 19 September 2018, hamsters were split into 8 groups of 7 males and 7 females and assigned to the various diets: ‘oat–sunflower’, ‘potato–sunflower’, ‘buckwheat–sunflower’, ‘maize–bean’, ‘maize–pea’, ‘wheat–soybean’, ‘sugar beet’ and ‘sprouted wheat’. Groups were made to ensure similar body mass mean and variation (computed as SEM). Due to an insufficient number of animals, the ‘maize–pea’ group was only composed of 4 males and 4 females, and the ‘sugar beet’ group of 5 males and 5 females. Throughout the study, hamsters had *ad libitum* access to water and to the food items constituting their respective diets. Hamsters received 150 g of each food item when cages were changed because they needed cleaning. Unlimited access to food was ensured by providing an additional 150 g of seeds or tubers whenever the remaining quantity of these foods approached 75 g (quantity evaluated visually twice a week). An exception was made for the ‘sprouted wheat’ group having *ad libitum* access to plain wheat grains and limited access to specific quantities of germinated wheat (germination: 7 days at 20°C; 10 g during winter, 30 g before reproduction and 50 g during reproduction distributed twice a week—such quantities allowing *ad libitum* access to germinated wheat). ‘Sugar beet’-fed hamsters were switched to a ‘wheat–soybean’ diet at the end of hibernation (14 March 2019). All food items provided to the hamsters were organically produced, except ‘sugar beet’, which was not organically cultivated in Alsace. Sugar beet, wheat and pea were directly purchased from local farmers. Other food items were purchased from CELNAT (Haute-Loire, France).

The study was performed in the CNRS breeding unit in Strasbourg, France. Housing conditions were similar to the ones provided in previous studies (Kletty, 2020; Tissier *et al.*, 2021) and for maintenance breeding. Hamsters were housed individually in regulatory rodent cages (medium 265 × 420 × 237-mm cages for males and large 380 × 590 × 257-mm reproduction-compatible cages for females) and randomly distributed in two rooms with controlled temperature. Ambient temperature was set at 10 ± 1°C from October to March (period later referred to as winter) and at 20 ± 1°C later on, after a 2-week gradual transition. Humidity ranged from 35 to 55%. Light exposure followed the natural photoperiod at the latitude of Strasbourg, France (48.58°N). Enrichment was provided with a PVC tube, paper towels and wooden fibres adequate for nest building.

### Diet nutritional analysis

Seeds and tuber samples were freeze-dried to constant mass to obtain a dry weight (DW). The dried samples were crushed into a homogeneous powder with a RETSCH ZM200 grinder. Analyses were carried out in duplicate on samples weighed to the nearest 0.1 mg. Before analyses, samples were freeze-dried again to eliminate any remnant traces of water. Energy content was determined on 1-g pellets using a Parr 6200 calorimeter with benzoic acid as an external standard. Total lipids were determined on 1-g samples using a chloroform:methanol (2:1, v/v) solution as extraction solvent. Protein content was obtained from the reference website Feedipedia (Heuzé *et al.*, 2017). Mineral content was measured by complete calcination of 1-g samples at 450°C for 24 h. Carbohydrate content was estimated as the remaining part in sample constitution.

### Nutritional intake monitoring

During hibernation (19 September 2018 to 13 March 2019), the quantity of each food item ingested by each hamster (i.e. food consumption) was measured by collecting leftover food in the cages. The intervention was only carried out when changing the cage for cleaning if and only if the animal was active, i.e. during the inter-bout euthermia phases, in order to minimize disturbance. (1–2 times for males and 0–1 time for females during winter). Food consumption was also measured before reproduction (26–29 April 2019); a shorter duration chosen to avoid the effect of post-hibernation physiological processes (body condition or gonadal regeneration) on food consumption. Food consumption was obtained by deducting the mass of food leftovers from the mass of food given. The mass of food leftovers was estimated from a manually sorted dehydrated sample of the homogenized cage content (~10% of total cage content, except for tubers whose dehydrated remnants were fully sorted). All mass measurements were done to the nearest 0.1 g. Hamsters’ energy (J) and nutritional (g) intakes were obtained by multiplying tuber or grain nutritional content (in J/g and g/g of dry mass) with hamsters’ food consumption (in g converted to dry mass).

### Hibernation monitoring

During winter, torpor patterns were monitored by equipping hamsters with intraperitoneal iButton temperature loggers (ref. DS1922L, Maxim Integrated) coated in biocompatible bee wax. Chirurgical procedures were performed following the protocol described by Weitten *et al.* (2018). Due to a limited availability of iButtons, we were only able to implant these devices in 42 females, which implies that in each group, one or even two females for larger groups were not implanted. The choice was made randomly. All females for the sprouted wheat group were implanted, because we were unsure what to expect regarding the effects of germination on hibernation. Body temperature was recorded every 75 min at a resolution of 0.0625°C. Hamsters sometime express daily shallow torpors outside of hibernation (Shankar *et al.*, 2023). Thus, hibernation analysis was focused on the multi-day deep torpor bouts that were defined as periods of >24 h spent below a body temperature threshold of 20°C. Hibernation characteristics (hibernation duration and emergence date, number and duration of torpor bouts, total time spent in torpor and in euthermia) were computed from torpor patterns. Hibernation of each hamster was defined as the number of days between beginning of the first (immergence) and the end of the last (emergence) registered torpors. Individual body mass was measured before and after hibernation (19 September 2018 and 13 March 2019, respectively). Mass variation through winter was defined as the difference between these two dates.

### Reproductive trials

Reproductive pairs were formed with males and females from the same groups using the ZooEasy software with a maximum inbreeding threshold of 6%. Males’ reproductive capability was evaluated through testis size as described by Masson-Pévet *et al.* (1994). Testes were measured to the nearest 0.1 mm using a calliper (mean of 3 measures per testis). Females’ reproductive capability was assessed by checking vaginal orifice openness (categorized as either closed or open). Reproductive trials were conducted following the breeding unit protocol described by Tissier *et al.* (2017). Reproductive pairs were placed in clean 380 × 590 × 257-mm cages and provided with individual PVC refuge boxes as well as several feeding and drinking sources. Hamsters were weighed before and after reproductive trials. The first reproductive trials were conducted from 29 April 2019. Reproductive pairs were separated after 2 weeks and females were monitored for 20 days (maximal gestation duration). Females received an extra daily protein supply through earthworms following Tissier *et al.* (2017) protocol, though worm supplementation was reduced from 5 to 2 g. If no parturition occurred, a new reproductive trial was initiated with a second male, genetically unrelated to the first one (at the beginning of June, for an 8-day reproductive period; males’ genetic proximity assessed using ZooEasy). If a parturition occurred, pups and mother were weighed weekly, until separation from the mother at 5 weeks. During that time, earthworm daily supplementation was increased each week by 0.5 g per pup.

### Statistical analysis

As mammals, hamsters provide maternal care and females are thus the limiting factor in reproductive efficiency (Speakman, 2008). Therefore, the results presented below focused on females. Statistical analyses were performed using R (version 4.3.1; R Core Team, 2023). Principal component analysis was performed using the FactoMineR package (version 2.9; Le *et al.*, 2008). Effects were tested using linear models (lm) if the model fitted with parametrical analysis conditions. In this case, a Tukey *post hoc* test was then conducted. If parametrical models were inappropriate, non-parametrical qualitative Kruskall–Wallis (KW) or quantitative Mann–Whitney (MW) approaches were used, followed by Dunn’s *post hoc* test if relevant.

Hibernation characteristics were analysed using both a qualitative simple linear model and a multi-factorial model including energy, lipid and protein intakes, as well as total food consumption. Corrected Akaike information criterion (AICc) based model selection was used to determine relevant effects and identify collinearity between factors. This was done using the dredge function from the MuMIn package (version 1.47.5; Bartoń, 2023). Model selection was also used to identify key factors impacting body mass at the end of winter. In that case, multi-factorial analysis was performed using energy, lipid, protein and food consumption as well as the time spent in torpor and emergence date. For reproductive traits, models were constrained by low parturition occurrence. Parametrical models were inadequate (non-Gaussian distribution of linear models’ residuals, overdispersion of Poisson generalized linear models and effectives unfitting both classic and zero-inflated negative binomial models). Thus, for reproduction analyses, a non-parametrical approach was favoured. It was carried out by testing the factors impacting the chance of a litter (binomial approach), the number of born pups (quantitative approach), the pups’ survival (binomial approach) and the pups’ mass at weaning (quantitative approach).

**Table 1:** Female hamster food preferences during winter and before hibernation

Diet		Proportion of first and second item intake in total dry mass intake		Preference shift
First item	Second item	Winter Means ± SEM MW, *P* =	Pre-reproduction Means ± SEM MW, *P* =	MW, *P* =
Maize	Bean	85.0/15.0 ± 4.8% **0.001**	87.4/12.6 ±3.0% **0.001**	0.798
	Pea	41.7/58.3 ± 2.6% **0.021**	38.4/61.6 ± 9.2% 0.281	0.999
Wheat	Soybean	27.6/72.4 ± 3.4% **0.001**	20.3/79.7 ± 3.8% **0.001**	0.259
Sunflower	Potatoes	44.7/55.3 ± 3.6% 0.682	64.3/35.7 ± 10.9% **0.020**	**0.026**
	Oat	28.0/72.0 ± 1.8% **0.001**	8.7/91.3 ± 3.0% **0.001**	**0.002**
	Buckwheat	33.0/67.0 ± 2.0% **0.001**	16.4/83.6 ± 5.7% **0.001**	0.053

Female hamster food preferences indicated as the proportion of the main item over total food consumed (in dry mass) during winter (from 19 September 2018 to 13 March 2019) and before reproduction (from 26 to 29 April 2019). Values are means ± SEM with *P-*values showing statistical divergence from a random selection of 50% (MW, *P* < 0.05). Last column indicates whether a change in preference between the two periods is statistically significant. Statistically significant differences are indicated by bold *P-*values (MW, *P* < 0.05).

## Results

### Diet grouping

Preliminary statistical tests showed that ‘sunflowers’ groups (supplemented with ‘potatoes’, ‘oat’ or ‘buckwheat’) exhibited similar results in terms of energy, lipid, carbohydrates and protein intakes (lm, *P* > 0.60), hibernation behaviour (lm, *P* = 0.967), winter body mass variation (lm, *P* = 0.722) and reproductive outputs (lm, *P* = 0.575). Similarly, ‘maize’ groups (supplemented with ‘peas’ or ‘beans’) showed similar results regarding these parameters (lm, *P* < 0.45, *P* = 0.997, 0.190, 0.695). Therefore, those diets were respectively grouped as ‘supplemented sunflower’ and ‘supplemented maize’ diets. For more details, grouped diets were represented by different dot shapes on figures.

### Hamsters’ nutritional intakes

Females’ daily food (in dry mass) and energy intakes during winter did not vary between groups (KW, *P* = 0.391 & 0.805, respectively; See Supplementary Table S2a). However, differences between groups were observed for lipid (KW, *P* < 0.001), protein (KW, *P* = 0.003) and carbohydrate intakes (KW, *P* < 0.001). Lipid intake was statistically higher in the ‘supplemented sunflower’ group (Dunn, *P* < 0.015), and intermediate in the ‘wheat–soybean’ group (Dunn, *P* < 0.048). ‘Wheat–soybean’ hamsters also exhibited a higher protein intake (Dunn, *P* < 0.007). The three other groups had similar lower lipid and protein intakes (Dunn, *P* > 0.11), that were compensated by a higher carbohydrate intake (Dunn, *P* < 0.013).

Females’ daily food intakes (in dry mass) significantly increased before reproduction compared to hibernation (MW, *P* < 0.020; See Supplementary Table S2b). Before reproduction, we observed differences between groups regarding food dry mass (KW, *P* < 0.001), energy (KW, *P* = 0.003), carbohydrate (KW, *P* < 0.001), lipid (KW, *P* < 0.001) and protein (KW, *P* < 0.001) intakes. Food and energy intakes were higher for the ‘sprouted wheat’ group (Dunn, *P* < 0.041). This group also had a statistically higher carbohydrate intake (Dunn, *P* < 0.014) than other groups, except the ‘supplemented maize’ one (Dunn, *P* = 0.111). The ‘supplemented sunflower’ groups had the highest lipid intake (Dunn, *P* < 0.029), whilst the highest protein intake was observed for the ‘wheat–soybean’ (original and following ‘sugar beet’) and ‘sprouted wheat’ groups (Dunn, *P* < 0.021).

### Hamsters’ food selection

Hamsters’ food selection was studied based on their dry mass intake, which better reflected nutritional intake as tubers have a much higher water content than seeds (see Supplementary Table S1). In all groups, hamsters preferred one item over the other during at least one period (proportion of consumption significantly differed from 50%; MW, *P* < 0.005; See Table 1). Maize was largely favoured over beans whilst peas were only slightly avoided. Hamsters tended to consume much more soybean than wheat. Potato preference was variable, whilst other ‘supplemented sunflower’ hamsters favoured oat and buckwheat over sunflower. A difference in food preferences between hibernation and before reproduction was only observed in the ‘supplemented sunflower’ groups. Hamsters from these groups lowered even further their sunflower intake in favour of ‘oat’ for reproduction*,* whilst they increased sunflower intake over ‘potatoes’ (MW, *P* < 0.05).

### Winter

#### Torpors and nutritional intakes during hibernation

**Figure 1:**
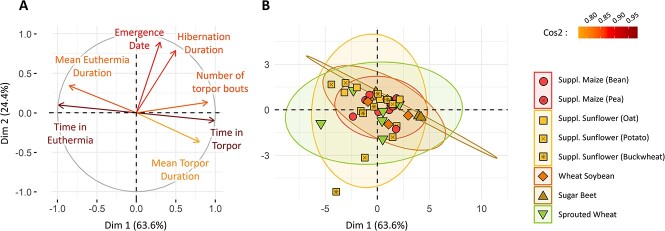
PCA characterizing hibernation. (A) Correlation between the various factors describing females’ hibernation and the two first axes of the PCA. Vector colours indicate how the corresponding factor correlates with the first two PCA axes (cos2 indicator). (B) Individuals repartition over the PCA, with the groups indicated by colours and shapes. Ellipses represent the groups repartition in a 95% confidence interval.

Principal component analysis (PCA) was performed on variables characterizing hibernation (Fig. 1a). The PCA vectors showed a positive correlation between the total number of torpor bouts, the total time spent in torpor and the mean torpor duration (Fig. 1a). These factors were also negatively correlated to the total time spent in euthermia and the mean duration of euthermia events. Hibernation duration and emergence date were also negatively correlated, but less or not correlated to other factors. Overall, the total time spent in torpor and the emergence date were well represented in the two first PCA dimensions (high cos2), and showed low covariance (orthogonal vectors). They were therefore used to characterize females’ hibernation in the following analysis.

Group distribution over the PCA was depicted as 95% confidence interval on Fig. 1b. Group distribution over the PCA was large, thus suggesting that individuals’ time in torpor and emergence date were greatly variable inside groups. Indeed, in the ‘supplemented sunflower’ groups, e.g. mean individual torpor use ranged from 17.6 to 84.0 h per week. Moreover, group distribution over the PCA strongly overlapped, indicating no differences in hibernation parameters between groups. Statistical models showed no significant differences in emergence date between groups (lm, *P* = 0.527; see Supplementary Fig. S1b for details). No differences were observed regarding torpor use either (lm, *P* = 0.072) (see Supplementary Fig. S1a for details).

**Figure 2:**
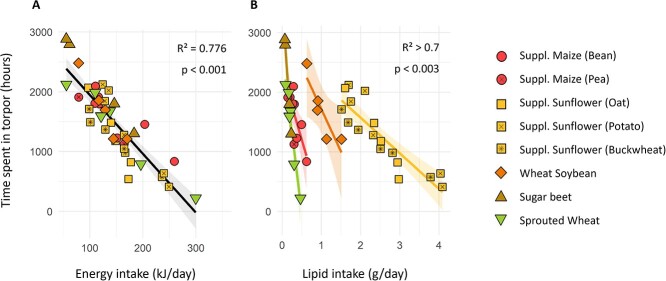
Total time spent in torpor (in hours) by females equipped with temperature loggers over winter, as a function of (A) energy and (B) lipid intake. Different groups are represented by different colours and shapes. Linear regression for all groups combined (A, in black) or per group when significantly different (lm, *P* < 0.05; B, coloured by group), with the confidence intervals (95%) shown by the grey areas (lm, *P* < 0.05). In Graph B, indicated values are the minimal R^2^ and maximal *P*-values.

Over winter, females from all groups spent a mean of 30 ± 20 h in torpor per week. Model selection based on relative AICc showed that the energy intake was the factor best predicting the time hamsters spent in torpor (lm, *P* < 0.001; Fig. 2a), with a negative correlation. The time spent in torpor was also negatively correlated with lipid intake inside groups (lm, *P* = 0.001; Fig. 2b), though this effect covaried with energy intake and was therefore not significant in models including both factors. Interestingly, though both lipids and energy effects were significant, energy intake itself was much more correlated to the hamsters’ overall consumption (cor = 0.904, *P* < 0.001) than to their lipid consumption (cor = 0.362, *P* < 0.001), thus explaining why lipid content variations in the diets did not induce food or energy intake differences between groups (Supplementary Table S2).

#### Body mass variation over winter

**Figure 3:**
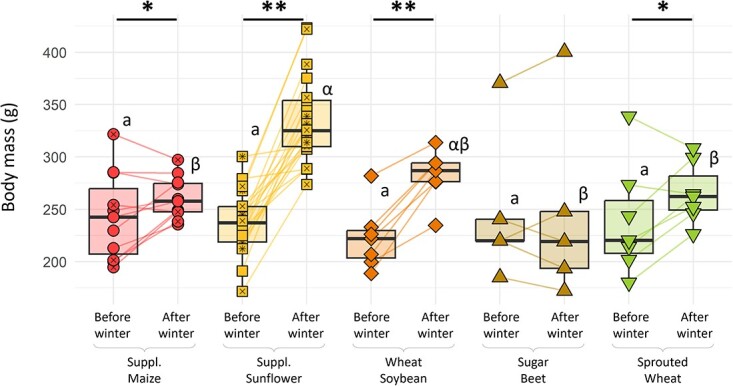
Females’ body mass before and after winter as a function of their diet. Different colours and shapes represent different groups. For each group, statistically significant differences between periods are indicated by asterisks (lm, *P* < 0.05). Statistically significant differences between groups (Tukey, *P* < 0.05) are indicated with different Latin letters before winter and Greek letters after winter.

Females from all groups started winter with a similar mean body mass of 237.4 ± 5.6 g (lm, *P* = 0.985). Females from the ‘supplemented sunflower’ group had a body mass of 333.4 ± 9.0 g at the end of winter, which was significantly higher than in the ‘supplemented maize’ group (260.7 ± 6.0 g, Tukey, *P* = 0.018), the ‘sugar beet’ group (246.7 ± 40.5 g, Tukey *P* = 0.001), the ‘sprouted wheat’ group (265.6 ± 11.0 g, Tukey, *P* = 0.004), but not the ‘wheat–soybean’ group (282.4 ± 9.3 g, Tukey *P* = 0.054); See Fig. 3). All other differences in body mass between groups were not significant (Tukey, *P* > 0.6). Females’ body mass before and after winter were correlated (i.e. bigger hamsters before winter were bigger at the end) (cor = 0.550, *P* < 0.001). This was considered by using the difference in body mass over winter as a parameter in the following models. On average, hamsters from all the groups (Tukey, *P* < 0.05) except ‘sugar beet’ (Tukey, *P* = 0.999) exhibited a body mass gain over winter (See Fig. 3). In this ‘sugar beet’ group, 3 of the 5 females lost body mass and ended winter period with the three lowest body mass of all females (Fig. 3).

**Figure 4:**
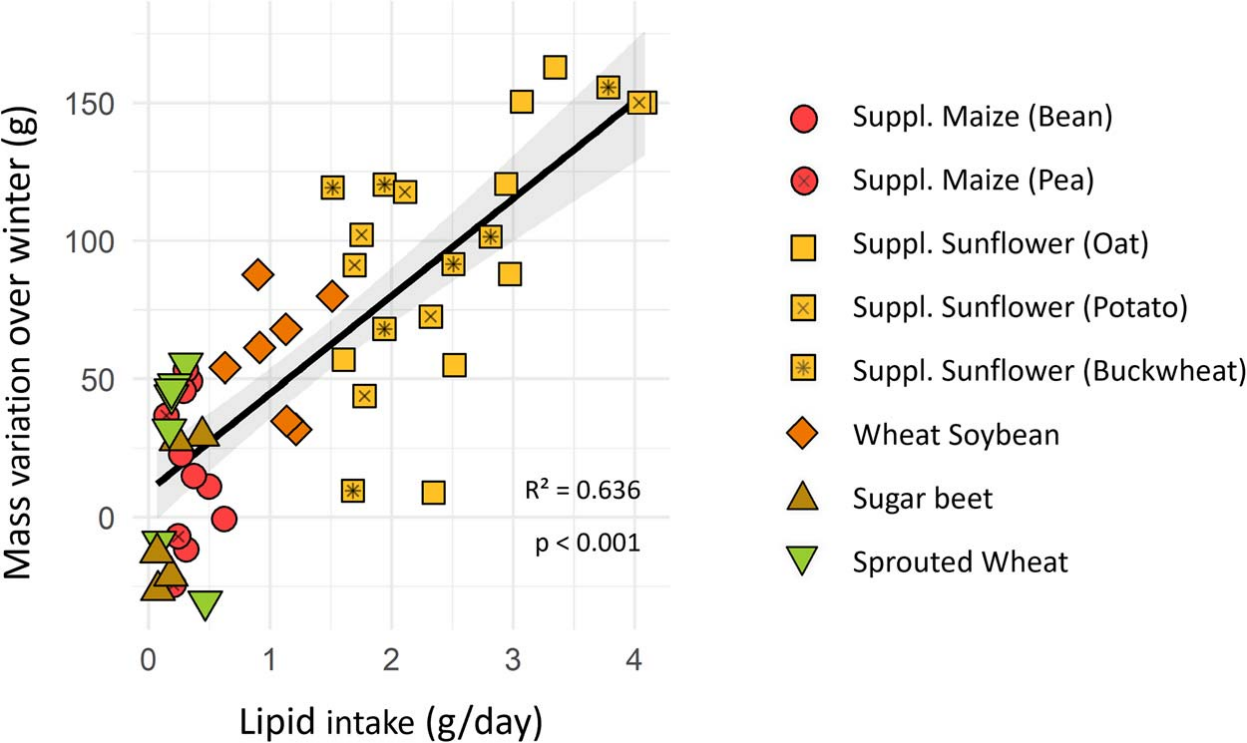
Body mass variation of females over winter as a function of their lipid intake. Different colours and shapes represent different groups. Linear regression for all groups combined is represented with the black line and the grey area represents its 95% confidence interval.

Females’ body mass gain over winter was diet dependant (lm, *P* < 0.001). Females from the ‘supplemented sunflower’ groups had a significantly higher gain than all other groups (Tukey, *P* < 0.025), except the ‘wheat–soybean’ one (Tukey, *P* = 0.148). Hamsters fed ‘wheat–soybean’ also had a significantly higher gain in body mass than those fed ‘sugar beet’ (Tukey, *P* = 0.003). All other differences in body mass variation over winter were not significant (Tukey, *P* > 0.07). After relative AICc-based model selection, the factor that correlated best with mass gain over winter was lipid consumption (lm, *P* < 0.001; Fig. 4), whilst proteins and carbohydrates had no effect (lm, *P* = 0.458 & 0.431). Hamsters spending more time in torpor gained less body mass (cor = −0.397, *P* = 0.009). This effect was indirect, as it was not significant (lm, *P* = 0.670) in models including lipid intake.

### Reproductive success

#### Birth rate

**Figure 5:**
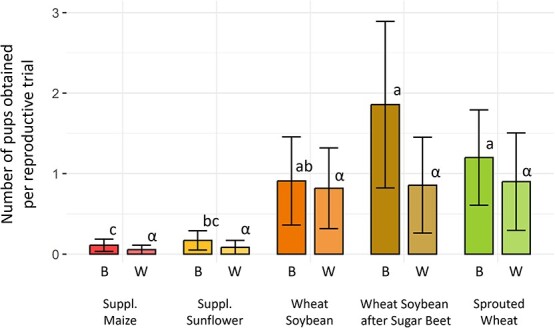
Number of pups at birth (B) and weaning (W) per reproductive trial. Colours represent different groups. Bars represent the mean number of pups ± SEM. Statistically significant differences between groups (Dunn, *P* < 0.05) are indicated with different Latin letters for birth and Greek letters for weaning.

Reproduction was low in all groups, with only 17% of reproductive trials leading to birth events. Eight litters (29 pups) were produced after the first reproductive trial and seven (16 pups) after the second. Litter size at birth ranged from 1 to 7 pups with a mean of 3.07 ± 1.86 pups per litter. Hamsters’ diet had a significant effect on the number of pups born per reproductive trial (KW, *P* = 0.017, See Fig. 5). The ‘supplemented maize’ and ‘supplemented sunflower’ groups exhibited a reproductive success close to zero born pups. On the opposite, ‘wheat–soybean following sugar beet’ had the more numerous born pups with 1.85 ± 1.00 pups per reproductive trials followed by the ‘sprouted wheat’ group. Those two groups differed significantly from ‘supplemented maize’ (Dunn, *P* = 0.022) and ‘supplemented sunflower’ (Dunn, *P* = 0.006). On the other hand, ‘wheat–soybean’ only differed from ‘supplemented sunflower’ (Dunn, *P* = 0.047) and none of the other groups (Dunn, *P* > 0.108).

**Figure 6:**
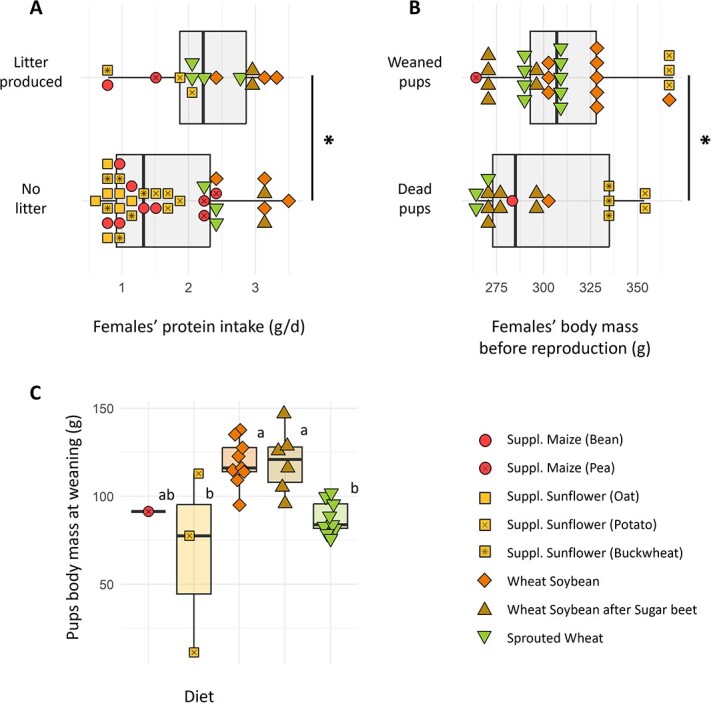
(A) Females having a litter or not as a function of protein consumption before reproduction. One point was excluded from the successfully breeding females due to an experimental accident preventing food consumption quantification (B) Pups’ survival as a function of mother’s body mass before reproduction. Asterisks indicate significant differences between groups (MW, *P* < 0.05). (C) Pups’ body mass at weaning as a function of the mother’s diet. Statistically significant differences between groups (Dunn, *P* < 0.05) are indicated with different letters.

At the beginning of the reproduction period, males had a mean mass of 410.8 ± 9.8 g. They all had well-developed testes (mean length of 18.2 ± 0.2 mm), whereas females’ vaginal orifices were all open, thus indicating that both were reproductively capable (Masson-Pévet *et al.*, 1994). Females were exposed to a second unrelated male when the first reproductive trial failed, thus avoiding a potential genetic defect in the first male. No link was observable between the number of newborns, their survival and mothers’ emergence date (MW, *P* = 0.567) or body mass variation through winter (MW, *P* = 0.872). Surprisingly, the females successfully having litters were the ones that spent more time in torpor (MW, *P* = 0.008) and had the lowest body mass at emergence (MW, *P* = 0.002). These females also had the lowest mass at the beginning of the reproduction trial, as the mass of females coming out of hibernation was strongly correlated with their mass at the time of reproduction (cor = 0.733, *P* < 0.001). These females had the highest protein intake before reproduction (Fig. 6a; KW, *P* = 0.043), but not during winter (KW, *P* = 0.139). No effect of carbohydrate nor lipid intakes was found on partition (KW, *P* = 0.476, 0.422).

#### Pups’ survival rate

There was no correlation between the number of pups per litter at birth and their survival at weaning (cor test, *P* = 0.211). The number of pups at weaning was similar in the ‘wheat–soybean’ (both original or following ‘sugar beet’) and ‘sprouted wheat’ groups (0.85 ± 0.55 pups; Dunn, *P* > 0.323). It was higher than in ‘supplemented maize’ and ‘supplemented sunflower’ groups (See Fig. 5), though the small number of litters did not permit to statistically confirm this tendency (KW, *P* = 0.061).

At an individual scale, a pup’s survival was not impacted by the time its mother had spent in torpor (MW, *P* = 0.536) nor by her emergence date (MW, *P* = 0.776) or body mass variation through winter (MW, *P* = 0.283). Independently of diets, pups’ survival was positively correlated with mothers’ body mass at the time of reproduction (KW, *P* = 0.009; Fig. 6b), but it was not statistically linked to mothers’ protein intake (KW, *P* = 0.088). Similarly, neither carbohydrate nor lipid intakes had an effect on survival (KW, *P* = 0.144, 0.484).

#### Pups’ body mass at weaning

Pups mass at weaning was diet dependant (KW, *P* = 0.002), as pups from both ‘wheat–soybean’ groups had a higher mass (119.5 ± 5.4 g) compared to the other groups (82.9 ± 3.1 g; Dunn, *P* < 0.010). Note that this difference was not significant with the ‘supplemented maize’ group since it was only composed of one survived pup (See Fig. 6c). Females’ body mass variation over lactation (from Week 1 to Week 5 post-parturition) was significantly impacted by the number of pups they weaned (KW, *P* < 0.001). Indeed, linear regression showed that females lost a mean of 20 g per pup fed through lactation (R^2^ = 0.733, KW, *P* < 0.001). This resulted for some females in a body mass loss of up to 87 g (34% of their total body mass) from Week 1 to Week 5 after parturition.

## Discussion

Overall, the tested diets induced contrasted effects along the hamsters’ seasonal cycle. These were linked with differences in nutrient intake. However, nutrient intakes were shaped by hamsters’ food selection and overall consumption, rather than diet quality, with a high variability inside the groups. This induced no group differences in term of hibernation behaviour, contradicting prediction (1.a). On the other hand, prediction (1.b) was verified, as hamsters from the lipid-rich groups (‘supplemented sunflower’ and ‘wheat–soybean’) exhibited a better body condition at the end of winter. Unexpectedly, hamsters with the best body condition at the end of winter had the worst reproductive success, opposing prediction (1.c). Reproductive success occurred especially in ‘wheat–soybean’ and, unexpectedly, in ‘sprouted wheat’. Nevertheless, the reproductive success was low in all our groups. Birth probability was higher for females that had a higher protein intake before reproduction, in accordance with prediction (2). Finally, pups’ survival was positively correlated with females’ body mass.

### Diet effects on hamsters’ hibernation and body condition

During winter, independently of diets, large inter-individual variations in food consumption were observed (e.g. 3–10 g/day in females from the ‘supplemented sunflower’ groups). However, a lower consumption of lipids was compensated by a higher carbohydrate intake in all hamsters. Therefore, energy intake was more dependent on hamsters’ overall food consumption than on diets’ nutritional characteristics. All diets enabled survival through winter, with no observed hibernation failure, and females even gained body mass during winter, with the exception of ‘sugar beet’-fed individuals. This showed that for all other groups, in an *ad libitum* food context, the tested diets fulfilled hamsters’ needs. Hibernators typically depend on body fat reserve to survive through winter, thus making energy savings through torpors critical (Humphries *et al.*, 2003; Bieber *et al.*, 2014; Siutz *et al.*, 2017). In those fat-storing species, body mass loss through hibernation is therefore the norm. Common hamsters exhibit a food-storing strategy, thus allowing them to rely on their food stock to meet their energy demands during winter (Nechay *et al.*, 1977). This strongly impacts the way hamsters manage their energetic balance and lowers their dependency on torpors (Humphries *et al.*, 2003).

In our study, large inter-individual variations within groups appeared in hibernation behaviour. Overall, hamsters with the highest energy intake spent less time in torpor, thus illustrating the energy balance commonly observed in hibernators (Humphries *et al.*, 2003) and previously described by Weitten *et al.* (2018) in common hamsters. On the other hand, food consumption was linked with body mass variation, allowing most individuals to gain mass over winter, even more so in the most lipid-rich diets. This is in accordance with previous studies in laboratory-housed (Siutz and Millesi, 2017) and free-ranging common hamsters (Siutz *et al.*, 2017) reporting a gain in body mass during winter and a high variability in hibernation behaviour. In laboratory conditions, these authors showed that torpor expression was linked to the size of food stores, since hamsters having access to 2000 g of food (sufficient to sustain prolonged euthermia) avoided torpors, whilst those having no stores but receiving 20 g of food per day performed some (Siutz and Millesi, 2017). Since some individuals did not express any torpor during winter, the common hamster can be considered as ‘facultative hibernator’. Such reports were also made in the eastern chipmunk, another food-storing hibernator, though the benefit on body condition of performing torpor versus being active and feeding was not reported (French, 2000). Nevertheless, all hamsters from our study performed torpor, even with *ad libitum* food access. Surprisingly, an increased use of torpor was linked to a better reproductive success, despite lower mass gain during hibernation. Thus, the use of torpor could be linked to benefits unrelated to energy saving, such as somatic maintenance (Giroud *et al.*, 2023) or reflect overwintering reproductive strategies that remain to be investigated in future studies.

‘Sugar beet’ cannot be considered as a favourable diet for winter, as females did not gain mass in this group. Sugar beet is particularly rich in carbohydrates, whilst other groups were much more balanced in macronutrients (Supplementary Table S1). Thus, through their stored food, hamsters had a very limited access to lipids, a nutrient that tends to limit body mass loss in hibernators (Dark, 2005). This imbalance might also impair carbohydrate metabolism, as hamsters showed signs of polyuria (observed through the faster soiling of cages). Such symptoms are often observed in hyperglycaemia-linked diseases in rodents (Xiao *et al.*, 2013). They could affect hibernation, as renal functions normally tend to decrease drastically during torpors (Zatzman, 1984). Additionally, hamsters in the lab tended to move and store tubers as small shavings that dried and degraded much faster than intact tubers. In the wild, it is therefore likely that sugar beet stocks would be depleted long before spring, thus reinforcing the idea that sugar beet is not favourable for hamsters’ hibernation.

Hamsters from the ‘supplemented sunflower’ and ‘wheat–soybean’ groups had the highest lipid consumption and gained more body mass. This result is coherent with the fact that lipids induce lower body mass loss through hibernation (Dark, 2005; Tissier *et al.*, 2021). This is due to a higher energy content in lipids, as well as beneficial physiological effects of some fatty acids (Dark, 2005). For example, polyunsaturated fatty acids (PUFAs) allow deeper and longer torpor bouts than saturated and monounsaturated fatty acids (Munro and Thomas, 2004). In the wild, this effect might be even more significant, as hamsters’ food hoards might be much more limited than in *ad libitum* laboratory conditions.

‘Supplemented maize’ and ‘sprouted wheat’ groups had much lower lipid intake but exhibited no mass loss through winter. On average, their energy intake was comparable with the one of hamsters fed with higher fat diets (‘supplemented sunflower’ and ‘wheat–soybean’ groups). Thus, hamsters adapted their food intake to compensate for the lower energy value of carbohydrates, in comparison with lipids, resulting in similar energy intake. A similar phenomenon has also been shown in other animal species (Bowen *et al.*, 1995; Mayntz *et al.*, 2009). Despite that, body mass gain during hibernation was lower compared to ‘supplemented sunflower’ groups, highlighting the benefits of lipids for hibernation. These ‘supplemented maize’ and ‘sprouted wheat’ diets can therefore be considered as viable, but perfectible diets for hamsters’ hibernation.

### Diet and body condition effect on reproduction

Reproductive outputs were contrasted, with ‘wheat–soybean’ (both following ‘sugar beet’ or not) and ‘sprouted wheat’ hamsters producing offspring (24 pups weaned for 19 females). On the other hand, the ‘supplemented maize’ and ‘supplemented sunflower’ groups produced few pups (4 pups weaned for 32 females), despite the legume seed provided with ‘maize’ and the high lipid content of ‘sunflower’. ‘Wheat–soybean’ provided relatively high amounts of both lipids and proteins, which explains the beneficial effect of this diet on reproduction, favouring both pups’ production and survival. On the other hand, only 36% of reproductive trials led to parturition events, with small litters of 3.6 ± 1.6 pups and a low survival at weaning of 68.5%. Though comparable to those reported by Tissier *et al.* (2021) in laboratory conditions, such reproductive rates are much lower than those reported in healthy wild populations by Surov *et al.* (2016). These cannot be attributed to our reproductive protocol, as it followed a standard one applied in our breeding unit with no reported decrease in reproductive efficiency. Thus, diet-induced limitations appear to be the main cause.

In the wild, even in the eventuality of food store consumption during reproduction, hamsters can benefit from nutritional diversification through fauna and sprouts of weeds and crops. On the other hand, seeds are known to be rich in anti-nutritional compounds, such as amylase or protease inhibitors (Singh *et al.*, 2023). Such compounds resorb during the germination process, thus increasing nutrient availability (Bau *et al.*, 1997; Zilic *et al.*, 2015). The lack of germinated items in the hamsters’ diets probably played a role in their reproductive failure. Such a mechanism might explain why the ‘sprouted wheat’ diet, despite its low protein content, gave results comparable to the ‘wheat–soybean’ groups in terms of reproductive efficiency. By contrast, diets based on wheat seeds induced reproductive failure in previous studies (Tissier *et al.*, 2017), even in association with protein-rich crops like pea (Kletty, 2020). Thus, germination might have increased nutrient bioavailability, turning ‘sprouted wheat’ into a viable diet. Similar observations were reported on rabbits, in which wheat germination increased sexual receptivity and reproductive success (Rodríguez-De Lara *et al.*, 2007). Nevertheless, ‘sprouted wheat’ pups grew more slowly, suggesting that mothers were still limited in some way. Sprouts have been observed in wild hamsters’ burrows (F.K. personal observation) indicating that such items are likely to be found in natural conditions during winter or spring. Thus, germinated items, which can be a diversity of crops, cover-crops or weeds, appear as important contributors to hamsters’ reproduction.

Reproductive success is a combination of two factors: 1) the number of pups produced and 2) their survival. In terms of pup production, females that ingested more proteins were more likely to have a litter. This is coherent with the fact that proteins have been identified as the reproduction limiting factor in several studies on hamsters (Tissier *et al.*, 2017; Weitten *et al.*, 2018) and other species (Bowen *et al.*, 1995; Speakman, 2008). The females who ingested the most protein did not necessarily belong to protein-rich diets as ‘supplemented maize’ diets. Indeed, higher protein intake was in fact induced by food selection (‘wheat–soybean’ groups) and/or higher overall food consumption (‘sprouted wheat’). This is in accordance with previous work in mammals showing a preference for regulation of protein intake over carbohydrate intake (Simpson and Raubenheimer, 1997). However, hamsters from the ‘supplemented sunflower’ groups tended to favour oat and buckwheat, especially before reproduction, thus lowering their intake in both proteins and lipids. The high amount of lipids in the protein-richer food may no longer make possible a food selection to increase protein intake. This highlights the need to carefully assess crop palatability when aiming at increasing nutrient availability in the hamsters’ environment. In that sense, ‘soybean’ appeared as the most promising tested legume, having the highest protein content and accounting for as much as 80% of the hamsters’ food consumption.

Surprisingly, females that produced offspring had the lowest body mass at the end of winter and at reproduction. This is contrasting with most studies, including on hamsters, that show positive correlations between body mass and reproductive success (Surov *et al.*, 2016; Tissier *et al.*, 2016; Bright Ross *et al.*, 2021). Two phenomena can explain the results of our study: 1) females with the highest body mass after hibernation belonged to the ‘supplemented sunflower’ groups, in which proteins were less abundant; 2) these females seemed to adapt their total food consumption to lipid or energy intake, resulting in lower food and protein intake than in other groups. This failure to adapt food intake to protein requirements has already been found in some species (Bowen *et al.*, 1995), but contrasting results have been reported for others (Mayntz *et al.*, 2009). The negative correlation between body mass and reproduction that we observed can therefore be considered as an indirect correlation induced by diets’ compositions rather than a direct effect. Such importance of protein intake during reproduction also explains the results observed in the ‘sugar beet’ group. When following a ‘sugar beet’ winter diet, ‘wheat–soybean’ appeared sufficient to allow pups’ production, even though females had the lowest body mass. However, pup survival was reduced in this group in comparison with hamsters fed a ‘wheat–soybean’ diet during hibernation. Indeed, females’ body mass had a positive effect on pups’ survival. This effect was expected, since lactation has a high energy cost and relies on the females’ energy reserves (Speakman, 2008; Bright Ross *et al.*, 2021). Females’ body mass loss through the lactation phase correlated with the number of pups at weaning. This suggests that pups’ survival was promoted by better female body condition allowing them to cope with the higher cost of raising more pups.

### Conclusion

As a food-storing species, the common hamster is able to hoard very large seed-based food reserves. Stores reported by Nechay *et al.* (1977) were even big enough to sustain hamsters’ needs for longer than their lifetime (Humphries *et al.*, 2003). Thus, seed- and tuber-based food stores are expected to be a significant part of the hamster diets during their yearly cycle, and not only during hibernation. Overall, hamsters’ hibernation and reproduction beneficiated from well-balanced seed diets, especially the ‘wheat–soybean’ diet. Lipids favoured a better hibernation with hamsters showing better body mass. This result is particularly interesting to promote hamster winter survival in the wild. However, even if female hamsters had better body condition before reproduction, this did not induce better reproductive success. Indeed, other factors, especially proteins, play a key role. Proteins can be provided in the environment of wild hamsters by implementing legumes (nitrogen fixing crops). Their palatability needs to be carefully assessed, as demonstrated by hamsters’ avoidance of beans. Thus, soybean appeared as the most promising legume in our study. The results obtained in the ‘sprouted wheat’ group suggest that increased nutrient bioavailability linked to germination can also allow to meet, at least in part, the nutritional needs of hamsters. Similarly, other nutrient sources in the wild could come from the fauna and weeds of agricultural fields, as shown by Tissier *et al.* (2019, 2018), which could be favoured by crop diversification and sustainable crop management.

The reproductive success of the ‘wheat–soybean’ following ‘sugar beet’ group underlined the interest of taking into account hamsters’ needs in crop rotation designs. Hamsters’ reproduction in the wild can start as early as April (Monecke and Wollnik, 2005). In most spring crops, this is earlier than crop sowing. Though late-growing crops are therefore beneficial for food storage when the crops reach maturity at the end of summer, this also means a lack of nutritional and protective cover at emergence from hibernation, a crucial time of the hamsters’ cycle. This highlights the need to enrich spring crops, like soybean. On the other hand, winter crops, such as wheat, provide a vegetal cover at burrow emergence, but are harvested earlier, usually when the second or third litters are born. Therefore, crop associations such as ‘wheat–soybean’*,* which can be grown in relay cropping, are promising to provide shelter and food to hamsters through their entire active season (Tanveer *et al.*, 2017).

Overall, these results illustrate the need to favour nutrient diversity in the diet of the common hamster, especially by providing proteins and lipid-rich storable items during their active season. This could be done by implementing well-diversified crops, though their palatability needs to be evaluated. These aspects also highly depend on the timing and management of the crops in the hamster’s environment. Thus, future agronomical studies should investigate how economically viable farming practises can provide proteins and lipids, but also vegetal cover and a diversified environment. Such considerations are certainly one of the ways that will enable the survival of this emblematic endangered species.

## Supplementary Material

Web_Material_coae082

## Data Availability

The data underlying this article will be shared on reasonable request to the corresponding author.
