# Supplementary material for: The endangered common hamster needs proteins: identifying diversified crop-based diets improving hibernation and reproductive success
Source: Conserv Physiol. 2024 Dec 13;12(1):coae082. doi: 10.1093/conphys/coae082 (PMC11638673; doi:10.1093/conphys/coae082)
Supplement: Web_Material_coae082 [file web_material_coae082.pdf]

Supplementary tables

Seed / Tubber	Water content (% of fresh mass)	Energy (kJ/g of dry mass)	Lipids (mg/g of dry mass)	Proteins (mg/g of dry mass)	Carbohydrates (mg/g of dry mass)	Minerals (mg/g of dry mass)
Maize	5.8 %	18.3	48.2	68.0	874.0	9.8
Bean	8.2 %	18.2	33.8	265.0	656.7	44.5
Pea	5.8 %	18.3	23.4	210.6	737.4	28.6
Wheat	5.0 %	18.3	28.5	136.2	818.8	16.5
Soybean	6.1 %	23.5	228.9	413.6	302.8	54.7
Sunflower	4.1 %	31.4	622.1	190.8	152.1	34.9
Buckwheat	9.7 %	18.4	38.9	140.4	800.3	20.4
Potatoes	77.4 %	16.6	25.0	108.0	820.7	46.2
Oat	7.9 %	18.8	52.2	139.3	790.8	17.7
Sugar beet	64.9 %	16.5	20.6	78.0	877.4	24.0

Supplementary Table 1: Nutritional content of tested crops. Water content is expressed as mass proportion over the total fresh sample mass (FM). Energy, lipids, proteins, carbohydrates and minerals are expressed in mass proportion per unit of dry sample mass (DM).

A

**Winter** females	Food intake (dry mass, g/d)	Energy intake (kJ/d)	Lipid intake (g/d)	Protein intake (g/d)	Carbohydrates intake (g/d)
Supplemented Maize<br>Protein rich diets	7.81 ± 0.85 [a]	142.7 ± 15.3 [a]	0.33 ± 0.04 [c]	0.91 ± 0.13 [b]	6.40 ± 0.67 [a]
Supplemented Sunflower<br>Lipid rich diets	6.19 ± 0.39 [a]	158.1 ± 10.1 [a]	2.51 ± 0.18 [a]	1.00 ± 0.06 [b]	2.19 ± 0.14 [b]
Wheat-Soybean<br>Balanced diet	6.12 ± 0.54 [a]	135.7 ± 12.1 [a]	1.06 ± 0.11 [b]	2.07 ± 0.20 [a]	2.75 ± 0.28 [b]
Sugar Beet<br>Carbohydrate rich diet	6.50 ± 2.20 [a]	159.6 ± 53.9 [a]	0.20 ± 0.07 [c]	0.76 ± 0.26 [b]	8.51 ± 2.87 [a]
Sprouted Wheat<br>Germinated Diet	7.97 ± 1.59 [a]	148.7 ± 29.6 [a]	0.23 ± 0.05 [c]	1.11 ± 0.22 [b]	6.66 ± 1.32 [a]

B

**Before Reproduction** females	Food intake (dry mass, g/d)	Energy intake (kJ/d)	Lipid intake (g/d)	Protein intake (g/d)	Carbohydrates intake (g/d)
Supplemented Maize<br>Protein rich diets	11.85 ± 0.59 [b]	217.41 ± 10.71 [b]	0.48 ± 0.03 [d]	1.45 ± 0.18 [b]	9.71 ± 0.45 [ab]
Supplemented Sunflower<br>Lipid rich diets	6.91 ± 0.75 [c]	186.61 ± 15.85 [b]	3.05 ± 0.17 [a]	1.16 ± 0.10 [b]	2.45 ± 0.47 [c]
Wheat-Soybean<br>Balanced diet	8.39 ± 0.40 [bc]	188.78 ± 9.10 [b]	1.58 ± 0.10 [b]	3.00 ± 0.17 [a]	3.41 ± 0.23 [c]
Wheat-Soybean<br>following Sugar Beet	9.33 ± 0.59 [bc]	204.32 ± 10.89 [b]	1.55 ± 0.02 [bc]	3.05 ± 0.09 [a]	4.36 ± 0.47 [bc]
Sprouted Wheat<br>Germinated Diet	28.58 ± 0.67 [a]	318.03 ± 12.19 [a]	0.50 ± 0.02 [cd]	2.37 ± 0.09 [a]	14.23 ± 0.55 [a]

Supplementary Table 2: Female hamster intakes in food (g dry mass / day), energy (kJ/d), lipids (g/d), proteins (g/d) and carbohydrates (g/d) during (A) hibernation (from 19 Sep 2018 to 13 Mar 2019), and (B) reproduction (from 26 Apr 2019 to 29 Apr 2019). Values are means ± SEM. Different letters indicate significant differences between values within a same column (Dunn, $p < 0.05$).

Supplementary figures

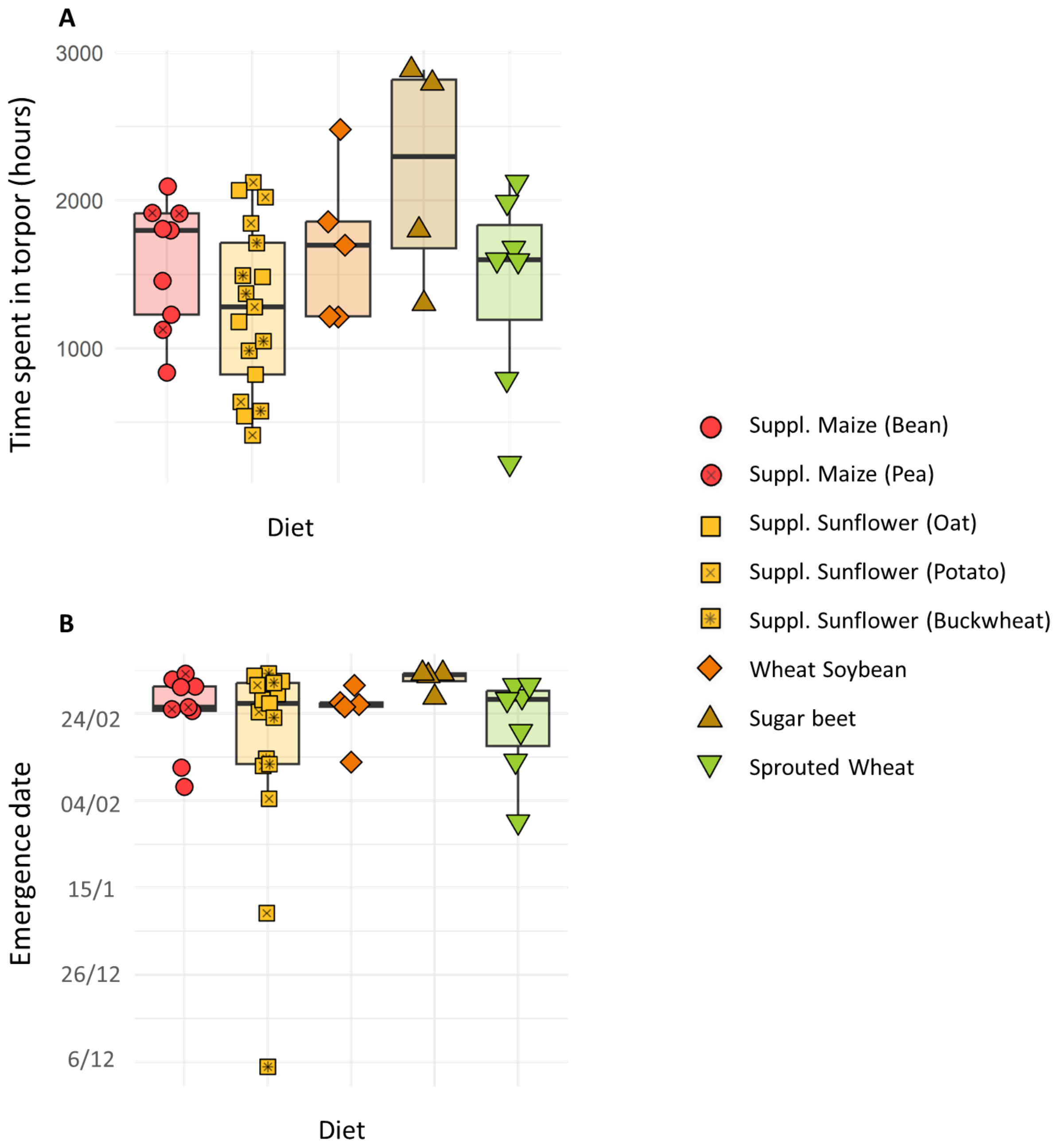

Supplementary figure 1:

(A) Time spent in torpor during females' hibernation (hours) and (B) female's date of emergence form last torpor (end of hibernation). Different colours and shapes represent different groups. Statistically significant group differences (Tukey, $p < 0.05$) are indicated with different letters.
